# Epistatic Effects on Abdominal Fat Content in Chickens: Results from a Genome-Wide SNP-SNP Interaction Analysis

**DOI:** 10.1371/journal.pone.0081520

**Published:** 2013-12-05

**Authors:** Fangge Li, Guo Hu, Hui Zhang, Shouzhi Wang, Zhipeng Wang, Hui Li

**Affiliations:** 1 Key Laboratory of Chicken Genetics and Breeding, Ministry of Agriculture, Harbin, People's Republic of China; 2 Key Laboratory of Animal Genetics, Breeding and Reproduction, Education Department of Heilongjiang Province, Harbin, People's Republic of China; 3 College of Animal Science and Technology, Northeast Agricultural University, Harbin, People's Republic of China; 4 Heilongjiang River Fishery Research Institute, Chinese Academy of Fishery Sciences, Harbin, People's Republic of China; Lund University, Sweden

## Abstract

We performed a pairwise epistatic interaction test using the chicken 60 K single nucleotide polymorphism (SNP) chip for the 11^th^ generation of the Northeast Agricultural University broiler lines divergently selected for abdominal fat content. A linear mixed model was used to test two dimensions of SNP interactions affecting abdominal fat weight. With a threshold of P<1.2×10^−11^ by a Bonferroni 5% correction, 52 pairs of SNPs were detected, comprising 45 pairs showing an Additive×Additive and seven pairs showing an Additive×Dominance epistatic effect. The contribution rates of significant epistatic interactive SNPs ranged from 0.62% to 1.54%, with 47 pairs contributing more than 1%. The SNP-SNP network affecting abdominal fat weight constructed using the significant SNP pairs was analyzed, estimated and annotated. On the basis of the network’s features, SNPs Gga_rs14303341 and Gga_rs14988623 at the center of the subnet should be important nodes, and an interaction between GGAZ and GGA8 was suggested. Twenty-two quantitative trait loci, 97 genes (including nine non-coding genes), and 50 pathways were annotated on the epistatic interactive SNP-SNP network. The results of the present study provide insights into the genetic architecture underlying broiler chicken abdominal fat weight.

## Introduction

Epistasis – the interactions between polymorphic loci, such as SNPs, genes or quantitative trait loci (QTLs) – is a hot topic in quantitative genetics [1]. Epistasis is a major factor that determines variation in quantitative phenotypes [2]. Mapping the epistatic loci affecting quantitative traits will advance our understanding of the genetic architecture of these complex phenotypes for humans and model organisms [2], [3]. Recently, the study of epistasis among genes, SNPs or QTLs has progressed rapidly [4]–[8], as has the study of epistasis in biological experiments [9].

Much progress has been made using genome-wide association studies (GWASs). Performing genome-wide SNPs interaction analysis represents the next step for detecting the variations of quantitative traits, because single-locus tests cannot identify the interactions among SNPs, genes or other genetic or environmental factors [10]. The markers detected by GWAS only explained a fraction of the heritable variance [11]–[13]. Identifying markers that show interactions would help to explain a higher proportion of the heritable variance. From sequence to phenotype, the SNP-SNP and gene-gene interactions can provide new insights into the genetic basis of complex traits.

In chickens (*Gallus gallus*), it has been suggested that epistatic interactions between genes (or QTLs) are important for quantitative traits such as growth and fatness traits [14]–[16]. Abdominal fat in meat-type chicken is a quantitative trait, and is an adverse economic factor; therefore, many QTLs and SNPs affecting fatness traits have been identified [17]–[21]. A previous study on chicken abdominal fat traits identified epistatic interactions among 10 candidate genes, and constructed networks of the interacting genes [15]. However, a large proportion of the effects of the epistatic interactions between polymorphic loci on fatness traits remain undetermined, and the whole genetic network is unclear. Hence, it is essential to study the effects of interactions between SNPs, genes and QTLs on fatness traits.

In this study, we aimed to determine the interactions between polymorphic loci affecting chicken abdominal fat weight (AFW). Pairwise epistatic interaction effects among genome-wide SNPs were detected. A network of significant SNP pairs was analyzed and annotated. The results provide further insight into the genetic network controlling abdominal fat deposition in chickens.

## Materials and Methods

### Ethics Statement

All animal work was conducted according to the guidelines for the care and use of experimental animals established by the Ministry of Science and Technology of the People’s Republic of China (Approval number: 2006–398) and was approved by the Laboratory Animal Management Committee of Northeast Agricultural University.

### Experimental Populations

The experimental chickens were from the Northeast Agricultural University broiler lines divergently selected for abdominal fat content (NEAUHLF) [22]. A total of 475 male individuals, 203 in the lean line and 272 in the fat line, derived from the 11^th^ generation population of NEAUHLF, were used in this study.

### SNP Genotyping

Genotyping was carried out using the Illumina Inc. (San Diego, CA, USA) chicken 60 K SNP chip, which contains 57,636 SNPs. Markers with minor allele frequencies less than 5% or monomorphic loci were filtered out. Individuals were removed who had 5% or more missing SNP genotypes. Finally, 45,611 SNPs in 475 individuals were used for the pairwise interaction analyses. SNP genotypes were provided by Zhang et al. [23].

### Genome-wide Pairwise Interaction Analysis

The EPISNP3 module in epiSNP_v4.2_Windows software package (Y. Da, Department of Animal Science, University of Minnesota, USA, http://animalgene.umn.edu/episnp/download.html) was used to test for epistatic effects [24].

The EPISNP’s statistical model for testing epistatic effects for AFW is
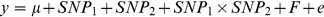
(1)


where *y* is the dependent variable (AFW), *μ* is the population mean, *SNP*_1_ and *SNP*_2_ are the two single-locus genotypic effects, *SNP*_1_×*SNP*_2_ is the two-locus interaction effect, *F* (family effect) is a random effect, and *e* is the random error.

The two-locus interaction effect was partitioned into four individual epistatic effects using the extended Kempthorne model, which allows Hardy-Weinberg disequilibrium and linkage disequilibrium (LD): Additive×Additive (AA), Additive×Dominant (AD), Dominant×Additive (DA), and Dominant×Dominant (DD) epistatic effects [24]. An F-test was used to test the significance of the two-locus interaction effect.

The P-value of model (1) was adjusted by a Bonferroni correction (4.16×10^9^; independent tests using the same data set, with a significance threshold of P<0.05), and the significance threshold of the test was determined as P<1.20×10^−11^.

### Contribution Rate Calculation

The contribution rate of every significant epistatic interactive SNP pair to phenotypic variation was calculated by the formula,
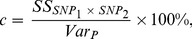
(2)where *SS_SNP_*_1_×*_SNP_*_2_ is the variance of the significant *SNP*_1_×*SNP*_2_ interactive effect (AA, AD, DA or DD), and *Var_p_* is the phenotype variance.

### SNP-SNP Network

The figure showing the SNP-SNP network with significant epistatic effects was drawn using the Cytoscape 2.8 software package [25]. The interactive SNPs affecting AFW with a pairwise interaction P-value of P<1.20×10^−11^ were loaded into Cytoscape to visualize the network. Based on entropy theory [26], the importance of the different subnets was evaluated by the formula,
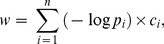
(3)where *w* is the importance of the subnet, *n* is the edge number (i.e. the number of SNP pairs), *p_i_* is the P-value of the interactive effect being tested for the i^th^ SNP pair, and *c_i_* is the contribution rate of the i^th^ SNP pair.

### Annotation of SNP-SNP Network

The purpose of this step was to annotate significant SNPs and mine QTLs or gene networks affecting abdominal fat traits. First, the significant interactive SNPs were put into the Chicken QTL database (http://www.animalgenome.org/cgi-bin/QTLdb/GG/i-ndex), and mapped in terms of QTLs. Second, the network was annotated with genes and pathways. A mean value of r^2^>0.8 [27] was observed in pairwise distances of approximately 0.2 Mb, as calculated by HAPLOVIEW v4.1 [28], and genes whose physical distance to the significant SNP was smaller than 0.2 Mb were selected. The positions of genes were acquired from UCSC Genome Bioinformatic site (http://genome.ucsc.edu/). Genes in the same network were put into the KEGG program (http://www.genome.jp/kegg/) to identify pathways that might affect the traits.

## Results

### SNP Makers

A total of 45,005 SNPs on 28 autosomes, the Z chromosome, linkage groups and 606 SNPs not assigned to any chromosomes in chickens were included in this study (Table 1). These markers covered 1026.23 Mb of the genome, with an average of 16.63 kb between adjacent markers.

**Table 1:** Summary of genome-wide markers.

GGA	SNPs’ number	GGA length (Mb)	Mean distance (kb)	GGA	SNPs’ number	GGA length (Mb)	Mean distance (kb)
**1**	7,143	200.95	28.13	**17**	844	10.61	12.57
**2**	5,299	154.46	29.15	**18**	845	10.89	12.89
**3**	4,081	113.65	27.85	**19**	804	9.89	12.30
**4**	3,314	94.16	28.41	**20**	1,460	13.92	9.53
**5**	2,172	62.23	28.65	**21**	726	6.88	9.48
**6**	1,714	35.84	20.91	**22**	295	3.89	13.19
**7**	1,770	38.17	21.56	**23**	577	6.02	10.43
**8**	1,394	30.62	21.97	**24**	676	6.23	9.22
**9**	1,168	24.02	20.57	**25**	170	2.02	11.88
**10**	1,297	22.42	17.29	**26**	617	5.03	8.15
**11**	1,196	21.87	18.29	**27**	472	4.84	10.25
**12**	1,324	20.45	15.45	**28**	563	4.46	7.92
**13**	1,128	18.32	16.24	**LEG22**	103	0.89	8.64
**14**	984	15.76	16.02	**LEG64**	2	0.005	2.27
**15**	1,010	12.93	12.80	**Z**	1,844	74.63	40.47
**16**	13	0.17	13.08	**UN** *	606	/	/

* These SNPs were not assigned to any chromosomes.

### Pairwise Interaction Effects Analysis

**Figure 1:**
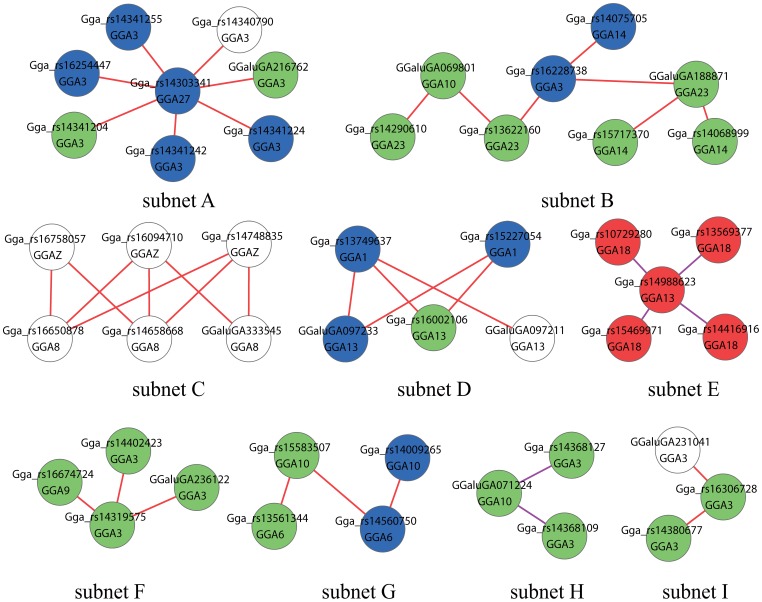
Epistatic network among SNPs affecting abdominal fat weight (AFW) in NEAUHLF. A node represents a SNP. The chromosome in which the SNP is located is shown in the circle. A pair of SNPs connected by an edge has a significant effect. The colors of the nodes represent P-values of the interaction (P<1×10^−13^ = red; P<1×10^−12^ = blue; P<1×10^−11^ = green; P<1×10^−10^ = white). The color of the edge indicates the epistatic effect type (AA = red; AD = purple).

EPISNP3 [24] was used to analyze the pairwise interaction effects among genome-wide SNPs for AFW across both lines. Fifty-two pairs of significant SNPs (Table 2), containing 45 pairs of AA and seven pairs of AD epistatic effects, were detected. No DA or DD epistatic effects were detected. The P-value of the epistatic effect between SNP Gga_rs13569377 on GGA18 (*Gallus gallus*, GGA) and Gga_rs14988623 on GGA13 reached 2.54×10^−14^, which was the most significant effect detected. There were six pairs of SNPs on the same chromosome (one pair on GGA2, four pairs on GGA3 and one pair on GGA10). The other 46 pairs of SNPs were distributed on different chromosomes. The contribution rate of the significant epistatic interactive SNP pairs ranged from 0.62% to 1.54%, with 47 pairs having a contribution rate of more than 1%. The 52 pairs of significant SNPs comprised 68 single SNPs covering 18 chromosomes. The position of each SNP is shown in Table S1. Many SNPs were detected to interact with the same locus; for example, seven SNPs on GGA3 all had epistatic interactions with Gga_rs14303341 on GGA27, and four SNPs on GGA18 interacted with Gga_rs14988623 on GGA13. The SNP pairs having the same SNPs are shown in Figure 1.

**Table 2:** Genome-wide significant pairwise epistatic interactive SNP pairs for abdominal fat weight, P<1.20×10^−11^.

GGAF^1^	SNP_1_ name	GGAS^1^	SNP_2_ name	Epistatic effect type	P-value	c (%)
0	GGaluGA194739	1	GGaluGA012915	AA	1.18×10^−11^	1.08
1	Gga_rs13866305	3	Gga_rs13717259	AD	4.90×10^−12^	0.68
1	Gga_rs13749637	13	Gga_rs16002106	AA	7.47×10^−12^	1.40
1	Gga_rs15227054	13	Gga_rs16002106	AA	8.52×10^−12^	1.38
1	Gga_rs13749637	13	GGaluGA097211	AA	1.02×10^−11^	1.44
1	Gga_rs13749637	13	GGaluGA097233	AA	7.50×10^−13^	1.54
1	Gga_rs15227054	13	GGaluGA097233	AA	8.57×10^−13^	1.51
1	GGaluGA060937	14	GGaluGA101229	AA	3.17×10^−12^	1.26
2	Gga_rs16026770	2	GGaluGA146662	AA	6.60×10^−12^	1.05
2	Gga_rs14214495	13	Gga_rs15683090	AA	9.31×10^−12^	1.14
3	Gga_rs14319575	3	Gga_rs14402423	AA	5.96×10^−12^	0.91
3	Gga_rs14380677	3	Gga_rs16306728	AA	8.25×10^−12^	1.10
3	Gga_rs16306728	3	GGaluGA231041	AA	1.09×10^−11^	1.07
3	Gga_rs14319575	3	GGaluGA236122	AA	5.96×10^−12^	0.91
3	Gga_rs14388313	5	Gga_rs14521876	AA	8.46×10^−13^	1.14
3	Gga_rs14368109	10	GGaluGA071224	AD	6.80×10^−12^	0.62
3	Gga_rs14368127	10	GGaluGA071224	AD	7.23×10^−12^	0.63
3	Gga_rs16228738	14	Gga_rs14075705	AA	8.12×10^−13^	1.42
3	Gga_rs16222762	20	Gga_rs14272866	AA	1.30×10^−12^	1.13
3	Gga_rs16228738	23	Gga_rs13622160	AA	7.99×10^−12^	1.20
3	Gga_rs16228738	23	GGaluGA188871	AA	9.80×10^−12^	1.15
3	Gga_rs14340790	27	Gga_rs14303341	AA	1.16×10^−11^	1.25
3	Gga_rs14341204	27	Gga_rs14303341	AA	1.42×10^−12^	1.29
3	Gga_rs14341224	27	Gga_rs14303341	AA	6.31×10^−13^	1.36
3	Gga_rs14341242	27	Gga_rs14303341	AA	5.68×10^−13^	1.38
3	Gga_rs14341255	27	Gga_rs14303341	AA	5.68×10^−13^	1.38
3	Gga_rs16254447	27	Gga_rs14303341	AA	6.31×10^−13^	1.36
3	GGaluGA216762	27	Gga_rs14303341	AA	1.36×10^−12^	1.31
4	Gga_rs15480969	20	Gga_rs14276105	AA	1.05×10^−11^	1.54
7	GGaluGA317680	14	Gga_rs15718248	AA	1.05×10^−11^	1.23
9	Gga_rs16674724	3	Gga_rs14319575	AA	1.05×10^−12^	1.01
10	Gga_rs15583507	6	Gga_rs13561344	AA	2.80×10^−12^	1.32
10	Gga_rs14009265	6	Gga_rs14560750	AA	5.35×10^−13^	1.18
10	Gga_rs15583507	6	Gga_rs14560750	AA	2.15×10^−12^	1.30
10	GGaluGA066690	10	GGaluGA066877	AA	4.66×10^−13^	1.27
10	GGaluGA069801	23	Gga_rs13622160	AA	5.56×10^−12^	1.15
10	GGaluGA069801	23	Gga_rs14290610	AA	4.64×10^−12^	1.26
14	Gga_rs14068999	23	GGaluGA188871	AA	3.29×10^−12^	1.12
14	Gga_rs15717370	23	GGaluGA188871	AA	3.29×10^−12^	1.12
18	Gga_rs10729280	13	Gga_rs14988623	AD	6.56×10^−14^	1.10
18	Gga_rs13569377	13	Gga_rs14988623	AD	2.54×10^−14^	1.14
18	Gga_rs14416916	13	Gga_rs14988623	AD	9.00×10^−14^	1.07
18	Gga_rs15469971	13	Gga_rs14988623	AD	2.92×10^−14^	1.10
Z	Gga_rs14748835	8	Gga_rs14658668	AA	1.14×10^−11^	1.20
Z	Gga_rs16094710	8	Gga_rs14658668	AA	1.14×10^−11^	1.20
Z	Gga_rs16758057	8	Gga_rs14658668	AA	1.14×10^−11^	1.20
Z	Gga_rs14748835	8	Gga_rs16650878	AA	1.11×10^−11^	1.20
Z	Gga_rs16094710	8	Gga_rs16650878	AA	1.11×10^−11^	1.20
Z	Gga_rs16758057	8	Gga_rs16650878	AA	1.11×10^−11^	1.20
Z	Gga_rs14748835	8	GGaluGA333545	AA	1.14×10^−11^	1.20
Z	Gga_rs16094710	8	GGaluGA333545	AA	1.14×10^−11^	1.20
Z	Gga_rs15991936	10	Gga_rs15589655	AA	8.33×10^−12^	1.28

1 GGA F = The first chromosome in the pairwise epistasis analysis; GGA S = The second chromosome in the pairwise epistasis analysis; AA = Additive×Additive effect, AD = Additive×Dominance effect, DA = Dominance×Additive effect, DD = Dominance×Dominance effect; P-value = P-value of the effect being tested; c = The contribution rate (%) of every significant epistatic interactive SNP pair.

### SNP-SNP Network Analysis

To investigate the complex mechanism of epistatic effects on AFW, a network of SNPs having epistatic interaction affecting AFW was constructed. SNP epistatic interaction subnets containing more than three nodes are shown in Figure 1.

The degree of nodes refers to the number of edges connecting it, and an edge stands for a two-way interaction. For example, the degree of SNP Gga_rs14988623 was 4, and the degree of SNP Gga_rs14303341 was 7 (Figure 1). SNP Gga_rs14303341 on GGA27 with the most two-way interactions, whose degree was the maximum, could be seen as the hub site of subnet A in the network of epistatic SNPs. SNP Gga_rs14988623 on GGA13 connected to four SNPs on GGA18 in subnet E was also a hub site. Subnet C contained eight edges, which was the maximum. The eight edges all happened between GGAZ and GGA8, so subnet C implies that that the interaction occurs between the two chromosomes. Interactions between two chromosomes were also hinted at by subnets A, D, E, F, G, and H. Interactions within the same chromosome were suggested by subnet I. Subnet B contained SNP pairs from four chromosomes, which was the largest number of chromosomes in any subnet.

Interestingly, in some subnets, the SNPs were adjacent to one another on the same chromosome. For example, in subnet A, seven SNPs on GGA3 were physically close to one another. This phenomenon was also observed for subnets C, D, E, G and H. Thus, there seemed to be a concentration of adjacent SNPs in these subnets. This indicated that the interaction takes place in the corresponding regions, especially for subnet C.

The importance of the subnets, as evaluated by formula (3), is shown in Table 3. The subnets with higher scores are more important. According to formula (3), subnets should get higher scores and be more important if they have more significant P-values, larger contribution rates and more edges. That a subnet is more important implied that the subnet contained more information on the SNP epistatic effect affecting AFW.

**Table 3:** Description of SNPs epistatic interaction network.

Subnet	Importance	Node number	Edge number	The greatest degree^1^	Structure^2^	GGA^3^
Subnet A	1.115	8	7	7	Tree graph	3, 27
Subnet B	0.961	8	7	3	Tree graph	3, 10, 14, 23
Subnet C	1.051	6	8	3	Loop graph	8, Z
Subnet D	0.837	5	5	3	Loop graph	1, 13
Subnet E	0.589	5	4	4	Tree graph	13, 18
Subnet F	0.325	4	3	3	Tree graph	3, 9
Subnet G	0.450	4	3	2	Tree graph	6, 10
Subnet H	0.139	3	2	2	Tree graph	3, 10
Subnet I	0.239	3	2	2	Tree graph	3

1 The greatest degree = The greatest number of nodes in the subnet;

2 Structure = Topological structure of the subnet;

3 Chromosomes = Chromosomes contained in the subnet.

### SNP-SNP Network Annotation

First, the significant epistatic interactive SNPs were mapped to QTLs affecting chicken AFW (Table S2). Twenty-four SNPs were located in 22 QTLs; the other SNPs showed no associations with any QTLs. Some of the 24 SNPs were located in the same QTL, such as seven SNPs from GGA3 that mapped to QTL 9418, 1958, and 11816; and three SNPs in GGAZ that mapped to QTL 2268 and 12633. The results for subnet A suggested that QTLs (9418, 1958, 11816) on GGA3 interacted with QTLs (11809, 11817) on GGA27. QTL 3353 on GGA1 might interact with QTL 12630 on GGA13, based on the result of subnet D.

Second, fragments of 0.4 Mb were defined, and the fragments’ centers were the 68 single SNPs in the significant epistatic interactive SNP pairs. The distances between some SNPs were small, and the fragments may overlap. A union set was used when the fragments overlapped. Regions were determined according to the fragments and union sets (Table 4). The 97 genes (including nine non-coding genes) in these regions are listed in Table 4. Information on protein coding genes is listed in Table S3.

**Table 4:** Regions and genes of the subnets shown in Figure 1.

Subnet	GGA	Start^1^	End^2^	SNP Set	Gene Set	
					Gene	Non-coding gene
A	27	911875	1311875	Gga_rs14303341	*GOSR2, GJC1, CCDC43, WNT3, NSF,* *EFTUD2*	Blank
	3	34826541	35226541	Gga_rs14340790	*PLD5, PIGM*	*MIR1784*
	3	35348152	35919953	Gga_rs16254447, Gga_rs14341204, Gga_rs14341224, Gga_rs14341242, Gga_rs14341255, GGaluGA216762	*RGS7, CHRM3*	
B	3	5408301	5808301	Gga_rs16228738	*OTOR*	Blank
	10	11303758	11703758	GGaluGA069801	*TMC3, IL16*	Blank
	14	622937	1033997	Gga_rs15717370, Gga_rs14068999	*PARN, PLA2G10, LITAF, EIF2AK1, CCZ1,* *OCM*	*MIR193B, MIR365-1*
	14	7120547	7520547	Gga_rs14075705	*PDPK1, UBE2I IL21R*	Blank
	23	2694945	3094945	Gga_rs13622160	*PTPRU*	*MIR1724*
	23	3181182	3832163	GGaluGA188871, Gga_rs14290610	*PAQR7, RRAGC, POU3F1, MTF1, MEAF6,* *STMN1*	Blank
C	8	28415409	28925822	GGaluGA333545, Gga_rs14658668, Gga_rs16650878	*FPGT, TNNI3K, CRYZ, LHX8, CCDC101*	Blank
	Z	59561642	60090315	Gga_rs14748835, Gga_rs16094710, Gga_rs16758057	Blank	Blank
D	1	31828151	32246400	Gga_rs13749637, Gga_rs15227054	*SLC16A7*	Blank
	13	14586787	15071343	Gga_rs16002106, GGaluGA097211, GGaluGA097233	*CXCL14, NEUROG1, H2AFY, PITX1, PCBD2, CAMLG, SAR1B*	Blank
E	13	6167549	6567549	Gga_rs14988623	*GABRG2, GABRA1*	Blank
	18	9895245	10688532	Gga_rs14416916, Gga_rs15469971	*TIMP2, CYTH1, PGS1, SOCS3, TK1, GIPR,* *P4HB, ARHGDIA, PCYT2, MAFG, NME1,* *TOB1, LUC7L3, ANKRD40, XYLT2, CD300A,* *CANT1, LRRC59*	*MIR1652, MIR1637*
	18	10776127	11280354	Gga_rs10729280, Gga_rs13569377	*GGA3, SUMO2, MRPS7, HN1, NUP85,* *KCTD2, GRB2,*	*MIR1580*
F	3	5425936	5825936	Gga_rs14319575	*OTOR*	Blank
	3	98046994	98587484	GGaluGA236122, Gga_rs14402423	*DDX1*	Blank
	9	16975422	17375422	Gga_rs16674724	*MFN1, PIK3CA*	Blank
G	6	1690195	2231763	Gga_rs13561344, Gga_rs14560750	*WAPAL, OPN4, BMPR1A, SNCG*	*MIR1579*
	10	12739689	13376210	Gga_rs15583507, Gga_rs14009265	*NTRK3*	Blank
H	3	62613708	63023180	Gga_rs14368109, Gga_rs14368127	*ROS1, VGLL2*	Blank
	10	13962468	14362468	GGaluGA071224	*ST8SIA2, RGMA*	*MIR1611*
I	3	76275164	76675164	Gga_rs14380677	*SYNCRIP, SNX14, TBX18*	Blank
	3	77845258	78245258	Gga_rs16306728	*FAM46A*	Blank
	3	79788812	80188812	GGaluGA231041	*IMPG1, MYO6, TMEM30A*	Blank

1 Start = The start of the region;

2 End = The end of the region.

Third, genes in every subnet were submitted to the KEGG database. The 97 genes were located in 50 pathways (Table S4). Some genes were located in the same pathway, for example *GRB2*, *PDPK1*, *PIK3CA* and *SOCS3* are in the insulin-signaling pathway; and *GRB2*, *PIK3CA*, *SOCS3* and *IL21R* are in the Jak-STAT-signaling pathway.

## Discussion

Abdominal fatness traits in chickens are important quantitative traits, and are under complex genetic control. Understanding the molecular mechanisms underlying them will help efficient growth selection in broiler chickens. In this study, analyses using genome-wide SNPs were performed to examine the genetic contribution of epistatic effects to the phenotypic variation of chicken abdominal fatness traits.

GWASs have identified many significant association markers. However, many studies showed that the identified markers explain only a fraction of the heritable variance [11]–[13]. This discrepancy has been ascribed to the insufficient power of GWAS to identify gene–gene interactions [13]. Zuk et al. [29] showed that the “missing” heritability of Crohn’s disease is 62.8%, and that genetic interactions could account for 80% of this missing heritability. Recently, most genome-wide association analyses in chickens have focused on individual SNP marker association analysis [20], [30]. However, quantitative traits often arise from the combined effects of multiple loci [14]–[16]. In the present study, the contribution rates of the significant epistatic interactive SNP pairs to AFW ranged from 0.62% to 1.54%. Two SNP pairs showed the largest contribution rate of 1.54%, and the contribution rates of 47 pairs were more than 1%. In addition, we calculated the contribution rates of single SNPs in the GWAS (Table S5). The single SNP Gga_rs14276105, which was significant in GWAS (Table S5), showed the largest contribution rate 1.46%. Comparing the results showed that the contribution rates of significant SNP pairs was larger than that of the single SNPs contained in the pairs. Although the contribution rates of the significant epistatic interactive SNP pairs might be overestimated for model (1), there were no high dimension interactions; therefore, we concluded that epistasis has an important effect on the phenotypic variance and cannot be ignored. Our results also showed that Gga_rs14276105 was the only SNP that was significant in an association study among the significantly interacting SNPs (Table S5). Wu et al. [31] also demonstrated that the majority of significantly interacting SNPs did not show marginal association in their data. This suggested that testing interactions of SNPs with only one significant locus association would lose the majority of interactions.

The features of scale-free networks are several nodes with large degrees and many nodes with few connections. Further, the nodes with large degrees should be the hub sites of the network. Our SNP-SNP network displayed the features of a scale-free network. The degree of one node was 7, and of another was 4; and 47 nodes had a degree of 1 in our SNP–SNP network. Thus, the nodes (Gga_rs14988623 and Gga_rs14303341) with larger degrees should be the important ones. Scale-free behavior has been demonstrated in many biological interaction networks, such as protein or gene interaction networks [32].

The formula for evaluating the importance of subnets proposed in the present study was based on the entropy theory [26]. The evaluation indices comprised the edge number, P-value and the contribution rate of every SNP pair in the subnets. The importance evaluation score stands for the information quantity of a subnet. A subnet containing more information would be more important. We suggested that subnets A and C are the most important; as they received the top two importance evaluation scores (Table 3).

Subnet A was radial, with seven SNPs connecting the center. A previous study concluded that many significant epistatic effects involving one locus were less likely to be random than was a single epistatic effect [33]; i.e., the network is less likely to be random. Combined with the results of the scale-free network analysis, we identified Gga_rs14303341 as the important SNP. The annotated information indicated that Gga_rs14303341 maps to QTLs (11809, 11817) [34], and the genes *GOSR2*, *GJC1*, *CCDC43*, *WNT3*, *NSF* and *EFTUD2* are within the defined 0.4 Mb region. These QTLs and genes should receive attention in a future study.

Subnet C was a loop graph, and contained six SNPs: three on GGAZ and three on GGA8. Between the two chromosomes, nine (3×3) pairs of SNP interactions were obtained, and eight of these interactions were significant. In addition, the SNPs were adjacent to one another on GGAZ and GGA8. The result indicated that the interaction occurs between the two regions on GGAZ (59,561,642–60,090,315) and GGA8 (28,415,409–28,925,822).

The epistatic interactions of SNPs might provide the basis for identifying interactions among genes, QTLs, or even pathways; therefore, we annotated the network. The result showed that 35% (24/68) of the SNP markers are located in regions containing 22 QTLs associated with chicken AFW (Table S2). The radial network based on subnet A suggested interactions between QTLs. A QTLs network with a similar topological structure was found in a study of chicken growth traits by Carlborg et al. [16]. They identified five significant epistatic QTL pairs that also formed a radial network and that accounted for a significant amount of the phenotypic variance.

Gene-by-gene interaction plays a major role in genetic studies of quantitative traits; however, the detection of gene–gene interaction has been traditionally assessed by SNP interactions. Single SNPs cannot capture the total variation of a gene; therefore, SNP interactions cannot represent gene–gene interactions. Thus, Cui et al. [35] and Li et al. [36] extended the idea of single SNP interactions to haplotype interactions, and proposed a novel statistical approach for capturing variations in genes and potential interactions between two genes. In the present study, we found an interesting phenomenon in some subnets, in which SNPs on the circumference were very near in physical distance. Therefore, we believe that the definition of block interaction may provide additional biological insights, compared with the analysis of SNP interactions.

In this study, we detected 26 regions on 12 chromosomes. Ninety-seven genes (including nine non-coding genes), including *BMPR1A*, *GIPR*, *GRB2*, *LITAF*, *SOCS3*, *WNT3* and *PDPK1*, were identified in these regions. Based on the literature, some genes are associated with obesity; for example, *BMPR1A* is associated with human obesity [37]. *GIPR* on GGA18 is associated with fat droplet formation [38]. *LITAF* plays an important role in the regulation of tumor necrosis factor alpha (TNF-α), which is important in adipose tissue [39], [40]. *WNT3* is part of the Wnt-signaling pathway, which is one of the pathways operating in fat cells [41]. *PDPK1* affects the fat-pad mass in mice [42]. We identified 50 pathways related to AFW, according to gene annotation analysis (Table S4). Some of the pathways are related to obesity, such as the Jak-STAT-signaling pathway and the insulin-signaling pathway [43], [44].

## Conclusions

Fifty-two pairs of significant epistatic interactive SNPs and their epistatic networks were determined. Two important SNPs and their interaction regions were proposed based on the network of significant epistatic interactive pairs. Relevant genes, pathways and QTLs were annotated. The results advanced our understanding of the genetic architecture of abdominal fatness traits, hinted at their molecular mechanisms, and will contribute to faster progress in the artificial genetic selection of broiler chickens.

## Supporting Information

Table S1 **SNPs contained in the significant SNP pairs.**
(DOC)

Table S2 **SNPs mapped to QTLs.** The QTL information was obtained from http://www.animalgenome.org/cgi-bin/QTLdb/GG/i-ndex.
(DOC)

Table S3 **Information on protein coding genes.** The information was obtained from http://www.genatlas.org/.
(DOC)

Table S4 **Genes in pathways.** Pathway information was obtained from http://www.genome.jp/kegg/.
(DOC)

Table S5 **Results of the genome-wide association study of significantly interacting SNPs.** The threshold of GWAS is P<2.7×10^−7^ by a Bonferroni 5% correction.
(DOC)
