# Supplementary material for: Epistatic Effects on Abdominal Fat Content in Chickens: Results from a Genome-Wide SNP-SNP Interaction Analysis
Source: PLoS One. 2013 Dec 5;8(12):e81520. doi: 10.1371/journal.pone.0081520 (PMC3855290; doi:10.1371/journal.pone.0081520)
Supplement: Table S1 — SNPs contained in the significant SNP pairs. (DOC) [file pone.0081520.s001.doc]

**Table S1.** **SNPs contained in the significant SNP pairs.**

| GGA | SNP | position(galGAL4) | GGA | SNP | position(galGAL4) |
| --- | --- | --- | --- | --- | --- |
| 0 | GGaluGA194739 |  | 8 | Gga_rs14658668 | 28710751 |
| 1 | Gga_rs13749637 | 32028151 | 8 | Gga_rs16650878 | 28725822 |
| 1 | Gga_rs15227054 | 32046400 | 9 | Gga_rs16674724 | 17175422 |
| 1 | GGaluGA012915 | 36726043 | 10 | GGaluGA066690 | 2965113 |
| 1 | Gga_rs13866305 | 51665390 | 10 | GGaluGA066877 | 3267117 |
| 1 | GGaluGA060937 | 184673552 | 10 | GGaluGA069801 | 11503758 |
| 2 | GGaluGA146662 | 49379304 | 10 | Gga_rs15583507 | 12939689 |
| 2 | Gga_rs16026770 | 66417173 | 10 | Gga_rs14009265 | 13176210 |
| 2 | Gga_rs14214495 | 86024956 | 10 | GGaluGA071224 | 14162468 |
| 3 | Gga_rs16228738 | 5608301 | 10 | Gga_rs15589655 | 16529144 |
| 3 | Gga_rs14319575 | 5625936 | 13 | Gga_rs15683090 | 5528536 |
| 3 | Gga_rs16222762 | 9192424 | 13 | Gga_rs14988623 | 6367549 |
| 3 | Gga_rs13717259 | 17185988 | 13 | Gga_rs16002106 | 14786787 |
| 3 | Gga_rs14340790 | 35026541 | 13 | GGaluGA097211 | 14827154 |
| 3 | Gga_rs16254447 | 35548152 | 13 | GGaluGA097233 | 14871343 |
| 3 | Gga_rs14341204 | 35579162 | 14 | Gga_rs15717370 | 822937 |
| 3 | Gga_rs14341224 | 35630493 | 14 | Gga_rs14068999 | 833997 |
| 3 | Gga_rs14341242 | 35682536 | 14 | Gga_rs15718248 | 1289989 |
| 3 | Gga_rs14341255 | 35699945 | 14 | GGaluGA101229 | 5235785 |
| 3 | GGaluGA216762 | 35719953 | 14 | Gga_rs14075705 | 7320547 |
| 3 | Gga_rs14368109 | 62813708 | 18 | Gga_rs14416916 | 10095245 |
| 3 | Gga_rs14368127 | 62823180 | 18 | Gga_rs15469971 | 10488532 |
| 3 | Gga_rs14380677 | 76475164 | 18 | Gga_rs10729280 | 10976127 |
| 3 | Gga_rs16306728 | 78045258 | 18 | Gga_rs13569377 | 11080354 |
| 3 | GGaluGA231041 | 79988812 | 20 | Gga_rs14272866 | 5641267 |
| 3 | Gga_rs14388313 | 83505275 | 20 | Gga_rs14276105 | 8502011 |
| 3 | GGaluGA236122 | 98246994 | 23 | Gga_rs13622160 | 2894945 |
| 3 | Gga_rs14402423 | 98387484 | 23 | GGaluGA188871 | 3381182 |
| 4 | Gga_rs15480969 | 4870857 | 23 | Gga_rs14290610 | 3632163 |
| 5 | Gga_rs14521876 | 21307724 | 27 | Gga_rs14303341 | 1111875 |
| 6 | Gga_rs13561344 | 1890195 | Z. | Gga_rs14748835 | 59761642 |
| 6 | Gga_rs14560750 | 2031763 | Z. | Gga_rs16094710 | 59861827 |
| 7 | GGaluGA317680 | 26291266 | Z. | Gga_rs16758057 | 59890315 |
| 8 | GGaluGA333545 | 28615409 | Z. | Gga_rs15991936 | 79335099 |
