# Supplementary material for: Epistatic Effects on Abdominal Fat Content in Chickens: Results from a Genome-Wide SNP-SNP Interaction Analysis
Source: PLoS One. 2013 Dec 5;8(12):e81520. doi: 10.1371/journal.pone.0081520 (PMC3855290; doi:10.1371/journal.pone.0081520)
Supplement: Table S2 — SNPs mapped to QTLs. The QTL information was obtained from http://www.animalgenome.org/cgi-bin/QTLdb/GG/i-ndex. (DOC) [file pone.0081520.s002.doc]

**Table S2.** **SNPs mapped to QTLs.** The QTL information was obtained from http://www.animalgenome.org/cgi-bin/QTLdb/GG/ i-ndex.

| QTL_ID | GGA | SNP | QTL_start | QTL_end | Significance |
| --- | --- | --- | --- | --- | --- |
| 3353 | 1 | Gga_rs13749637Gga_rs15227054GGaluGA012915Gga_rs13866305 | 23819028 | 64096915 | Suggestive |
| 6806 | 1 | Gga_rs13866305 | 49948878 | 52765543 | Suggestive |
| 12478 | 1 | Gga_rs13866305 | 49948878 | 52765543 | Suggestive |
| 7010 | 1 | GGaluGA060937 | 150057272 | 184976321 | Significant |
| 1845 | 1 | GGaluGA060937 | 184028842 | 185081596 | Suggestive |
| 9418 | 3 | Gga_rs16222762Gga_rs13717259Gga_rs14340790Gga_rs16254447Gga_rs14341204Gga_rs14341224Gga_rs14341242Gga_rs14341255GGaluGA216762 | 7519336 | 43742377 | Significant |
| 1941 | 3 | Gga_rs16222762 | 7519336 | 13908200 | Suggestive |
| 1947 | 3 | Gga_rs13717259 | 13908200 | 23678709 | Suggestive |
| 1958 | 3 | Gga_rs14340790Gga_rs16254447Gga_rs14341204Gga_rs14341224Gga_rs14341242Gga_rs14341255GGaluGA216762 | 23550758 | 48978327 | Suggestive |
| 12627 | 3 | Gga_rs16222762 | 7519336 | 13908200 | Significant |
| 11816 | 3 | Gga_rs14340790Gga_rs16254447Gga_rs14341204Gga_rs14341224Gga_rs14341242Gga_rs14341255GGaluGA216762 | 33595706 | 39878323 | Significant |
| 17303 | 5 | Gga_rs14521876 | 15794756 | 25113116 | Suggestive |
| 9432 | 5 | Gga_rs14521876 | 17345750 | 39496361 | Significant |
| 2076 | 5 | Gga_rs14521876 | 20214432 | 27151131 | Suggestive |
| 3320 | 5 | Gga_rs14521876 | 20214432 | 27151131 | Significant |
| 2167 | 7 | GGaluGA317680 | 23972409 | 35514724 | Suggestive |
| 2220 | 9 | Gga_rs16674724 | 11406093 | 22258179 | Suggestive |
| 12630 | 13 | Gga_rs16002106GGaluGA097211GGaluGA097233 | 7049258 | 16060614 | Suggestive |
| 11809 | 27 | Gga_rs14303341 | 1087600 | 3631192 | Suggestive |
| 11817 | 27 | Gga_rs14303341 | 1087600 | 3631192 | Significant |
| 2268 | Z | Gga_rs14748835Gga_rs16094710Gga_rs16758057 | 49302565 | 62384625 | Suggestive |
| 12633 | Z | Gga_rs14748835Gga_rs16094710Gga_rs16758057 | 49302565 | 62384625 | Suggestive |
