# Supplementary material for: Epistatic Effects on Abdominal Fat Content in Chickens: Results from a Genome-Wide SNP-SNP Interaction Analysis
Source: PLoS One. 2013 Dec 5;8(12):e81520. doi: 10.1371/journal.pone.0081520 (PMC3855290; doi:10.1371/journal.pone.0081520)
Supplement: Table S3 — Information on protein coding genes. The information was obtained from http://www.genatlas.org/. (DOC) [file pone.0081520.s003.doc]

**Table S3.** **Information on protein coding genes.** The information was obtained from http://www.genatlas.org/.

| GGA | Gene symbol | Gene_start | Gene_end | Full name |
| --- | --- | --- | --- | --- |
| 1 | *SLC16A7* | 32050439 | 32079281 | solute carrier family 16 |
| 3 | *OTOR* | 5580122 | 5585119 | otoraplin |
| 3 | *PLD5* | 34811264 | 34977620 | phospholipase D family |
| 3 | *PIGM* | 35211566 | 35213495 | phosphatidylinositol glycan anchor biosynthesis |
| 3 | *RGS7* | 35241157 | 35471485 | regulator of G-protein signaling 7 |
| 3 | *CHRM3* | 35727395 | 35981198 | cholinergic receptor, muscarinic 3 |
| 3 | *ROS1* | 62908209 | 62980553 | c-ros oncogene 1 , receptor tyrosine kinase |
| 3 | *VGLL2* | 62992150 | 62997528 | vestigial like 2 (Drosophila) |
| 3 | *SYNCRIP* | 76289509 | 76312354 | synaptotagmin binding, cytoplasmic RNA interacting protein |
| 3 | *SNX14* | 76326432 | 76373663 | sorting nexin 14 |
| 3 | *TBX18* | 76646415 | 76667486 | T-box 18 |
| 3 | *FAM46A* | 77866525 | 77870758 | family with sequence similarity 46, member A |
| 3 | *IMPG1* | 79802794 | 79856460 | interphotoreceptor matrix proteoglycan 1 |
| 3 | *MYO6* | 79860209 | 79930603 | myosin VI |
| 3 | *TMEM30A* | 80180050 | 80193546 | transmembrane protein 30A |
| 3 | *DDX1* | 98505119 | 98526380 | DEAD (Asp-Glu-Ala-Asp) box polypeptide 1 |
| 6 | *WAPAL* | 1737607 | 1793686 | wings apart-like homolog (Drosophila) |
| 6 | *OPN4* | 1829532 | 1852148 | opsin 4 |
| 6 | *BMPR1A* | 2035726 | 2074031 | bone morphogenetic protein receptor, type IA |
| 6 | *SNCG* | 2108598 | 2115737 | synuclein, gamma (breast cancer-specific protein 1) |
| 8 | *FPGT* | 28457546 | 28462055 | fucose-1-phosphate guanylyltransferase |
| 8 | *TNNI3K* | 28466299 | 28510637 | TNNI3 interacting kinase |
| 8 | *CRYZ* | 28533242 | 28538950 | crystallin, zeta (quinone reductase) |
| 8 | *LHX8* | 28607271 | 28618820 | LIM homeobox 8 |
| 8 | *CCDC101* | 28729100 | 28733359 | coiled-coil domain containing 101 |
| 9 | *MFN1* | 16984925 | 17004455 | mitofusin 1 |
| 9 | *PIK3CA* | 17020758 | 17039184 | phosphoinositide-3-kinase, catalytic, alpha polypeptide |
| 10 | *NTRK3* | 12720689 | 12898569 | neurotrophic tyrosine kinase |
| 10 | *ST8SIA2* | 13995207 | 14019205 | ST8 alpha-N-acetyl-neuraminide alpha-2 |
| 10 | *RGMA* | 14169409 | 14183205 | RGM domain family, member A |
| 13 | *GABRG2* | 6400934 | 6470500 | gamma-aminobutyric acid (GABA) A receptor, gamma 2 |
| 13 | *GABRA1* | 6533372 | 6575415 | gamma-aminobutyric acid (GABA) A receptor, alpha 1 |
| 13 | *CXCL14* | 14655190 | 14662456 | chemokine (C-X-C motif) ligand 14 |
| 13 | *NEUROG1* | 14676026 | 14677023 | neurogenin 1 |
| 13 | *H2AFY* | 14736402 | 14781620 | H2A histone family, member Y |
| 13 | *PITX1* | 14934514 | 14940878 | paired-like homeodomain 1 |
| 13 | *PCBD2* | 14971860 | 14989190 | pterin-4 alpha-carbinolamine dehydratase/ dimerization cofactor of hepatocyte nuclear factor 1 alpha (TCF1) 2 |
| 13 | *CAMLG* | 15032060 | 15035079 | calcium modulating ligand |
| 13 | *SAR1B* | 15065093 | 15071876 | SAR1 homolog B (S. cerevisiae) |
| 14 | *PARN* | 791607 | 826239 | poly(A)-specific ribonuclease |
| 14 | *PLA2G10* | 836324 | 846676 |  |
| 14 | *LITAF* | 924498 | 931316 | lipopolysaccharide-induced TNF factor |
| 14 | *EIF2AK1* | 958168 | 974389 | eukaryotic translation initiation factor 2-alpha kinase 1 |
| 14 | *CCZ1* | 1003428 | 1017262 |  |
| 14 | *OCM* | 1020715 | 1024105 |  |
| 14 | *IL21R* | 7297778 | 7310538 | interleukin 21 receptor |
| 14 | *PDPK1* | 7329857 | 7360340 |  |
| 14 | *UBE2I* | 7399021 | 7407204 | ubiquitin-conjugating enzyme E2I |
| 18 | *MIR1652* | 9906186 | 9906282 |  |
| 18 | *CANT1* | 10034761 | 10038436 | calcium activated nucleotidase 1 |
| 18 | *TIMP2* | 10040656 | 10047611 |  |
| 18 | *CYTH1* | 10062345 | 10070302 | cytohesin 1 |
| 18 | *PGS1* | 10096974 | 10115046 | phosphatidylglycerophosphate synthase 1 |
| 18 | *SOCS3* | 10116363 | 10118395 | suppressor of cytokine signaling 3 |
| 18 | *TK1* | 10142952 | 10144362 | thymidine kinase 1 |
| 18 | *GIPR* | 10181390 | 10184603 | gastric inhibitory polypeptide receptor |
| 18 | *P4HB* | 10195331 | 10202022 | prolyl 4-hydroxylase, beta polypeptide |
| 18 | *ARHGDIA* | 10205972 | 10214784 | Rho GDP dissociation inhibitor (GDI) alpha |
| 18 | *PCYT2* | 10223912 | 10238009 | phosphate cytidylyltransferase 2, ethanolamine |
| 18 | *MAFG* | 10240924 | 10242628 | v-maf musculoaponeurotic fibrosarcoma oncogene homolog G (avian) |
| 18 | *NME1* | 10253362 | 10256902 | non-metastatic cells 1, protein (NM23A) expressed in |
| 18 | *TOB1* | 10354078 | 10355907 | transducer of ERBB2, 1 |
| 18 | *LUC7L3* | 10393673 | 10406868 | LUC7-like 3 (S. cerevisiae) |
| 18 | *ANKRD40* | 10407625 | 10414278 | ankyrin repeat domain 40 |
| 18 | *LRRC59* | 10650718 | 10655016 | leucine rich repeat containing 59 |
| 18 | *XYLT2* | 10670852 | 10683456 | xylosyltransferase II |
| 18 | *CD300A* | 10684418 | 10688333 | CD300a molecule |
| 18 | *KCTD2* | 10900893 | 10907984 | potassium channel tetramerisation domain containing 2 |
| 18 | *HN1* | 10931323 | 10941484 | hematological and neurological expressed 1 |
| 18 | *SUMO2* | 10947883 | 10954469 |  |
| 18 | *NUP85* | 10960592 | 10972413 | nucleoporin 85kDa |
| 18 | *GGA3* | 10972741 | 10991646 | golgi-associated, gamma adaptin ear containing, ARF binding protein 3 |
| 18 | *MRPS7* | 10991674 | 10997091 | mitochondrial ribosomal protein S7 |
| 18 | *GRB2* | 11015583 | 11049321 | growth factor receptor-bound protein 2 |
| 23 | *PTPRU* | 2389910 | 2759580 | protein tyrosine phosphatase, receptor type, U |
| 23 | *STMN1* | 3198616 | 3201919 | stathmin 1 |
| 23 | *PAQR7* | 3204151 | 3207296 | progestin and adipoQ receptor family member VII |
| 23 | *RRAGC* | 3217149 | 3225969 | Ras-related GTP binding C |
| 23 | *POU3F1* | 3418910 | 3419428 |  |
| 23 | *MTF1* | 3502046 | 3514491 | metal-regulatory transcription factor 1 |
| 23 | *MEAF6* | 3708592 | 3714102 | MYST/Esa1-associated factor 6 |
| 27 | *GOSR2* | 1081553 | 1087770 | golgi SNAP receptor complex member 2 |
| *27* | *WNT3* | 1136138 | 1157180 |  |
| *27* | *NSF* | 1159104 | 1215306 | N-ethylmaleimide-sensitive factor |
| *27* | *CCDC43* | 1236371 | 1244170 | coiled-coil domain containing 43 |
| *27* | *GJC1* | 1272877 | 1276164 | gap junction protein, gamma 1, 45kDa |
| *27* | *EFTUD2* | 1287311 | 1302968 | elongation factor Tu GTP binding domain containing 2 |
