# Supplementary material for: Epistatic Effects on Abdominal Fat Content in Chickens: Results from a Genome-Wide SNP-SNP Interaction Analysis
Source: PLoS One. 2013 Dec 5;8(12):e81520. doi: 10.1371/journal.pone.0081520 (PMC3855290; doi:10.1371/journal.pone.0081520)
Supplement: Table S4 — Genes in pathways. Pathway information was obtained from http://www.genome.jp/kegg/. (DOC) [file pone.0081520.s004.doc]

**Table S4. Genes in pathways. Pathway information was obtained from http://www.genome.jp/kpegg/.**

| subnet | Gene | Pathway | Name |
| --- | --- | --- | --- |
| subnet A | *WNT3* | gga04340 | Hedgehog signaling pathway - Gallus gallus (chicken) |
|  |  | gga04916 | Melanogenesis - Gallus gallus (chicken) |
|  |  | gga04310 | Wnt signaling pathway - Gallus gallus (chicken) |
|  | *PIGM* | gga01100 | Metabolic pathways - Gallus gallus (chicken) |
|  |  | gga00563 | Glycosylphosphatidylinositol(GPI)-anchor biosynthesis - Gallus gallus (chicken) |
|  | *CHRM3* | gga04810 | Regulation of actin cytoskeleton - Gallus gallus (chicken) |
|  |  | gga04020 | Calcium signaling pathway - Gallus gallus (chicken) |
|  |  | gga04080 | Neuroactive ligand-receptor interaction - Gallus gallus (chicken) |
|  | *EFTUD2* | gga03040 | Spliceosome - Gallus gallus (chicken) |
|  | *GOSR2* | gga04130 | SNARE interactions in vesicular transport - Gallus gallus (chicken) |
| subnet B | *PLA2G10* | gga01100 | Metabolic pathways - Gallus gallus (chicken) |
|  |  | gga00592 | alpha-Linolenic acid metabolism - Gallus gallus (chicken) |
|  |  | gga04270 | Vascular smooth muscle contraction - Gallus gallus (chicken) |
|  |  | gga00565 | Ether lipid metabolism - Gallus gallus (chicken) |
|  |  | ga00591 | Linoleic acid metabolism - Gallus gallus (chicken) |
|  |  | gga00564 | Glycerophospholipid metabolism - Gallus gallus (chicken) |
|  |  | gga00590 | Arachidonic acid metabolism - Gallus gallus (chicken) |
|  | *PDPK1, RRAGC* | gga04150 | mTOR signaling pathway - Gallus gallus (chicken) |
|  | *PDPK1* | gga03320 | PPAR signaling pathway - Gallus gallus (chicken) |
|  |  | gga04910 | Insulin signaling pathway - Gallus gallus (chicken) |
|  |  | gga04510 | Focal adhesion - Gallus gallus (chicken) |
|  | *EIF2AK1* | gga05168 | Herpes simplex infection - Gallus gallus (chicken) |
|  |  | gga04141 | Protein processing in endoplasmic reticulum - Gallus gallus (chicken) |
|  |  | gga05164 | Influenza A - Gallus gallus (chicken) |
|  | *PARN* | gga03018 | RNA degradation - Gallus gallus (chicken) |
|  | *IL21R* | gga04060 | Cytokine-cytokine receptor interaction - Gallus gallus (chicken) |
|  |  | gga04630 | Jak-STAT signaling pathway - Gallus gallus (chicken) |
|  | *UBE2I* | gga04120 | Ubiquitin mediated proteolysis - Gallus gallus (chicken) |
|  |  | gga03013 | RNA transport - Gallus gallus (chicken) |
|  | *STMN1* | gga04010 | MAPK signaling pathway - Gallus gallus (chicken) |
| subnet C | *FPGT* | gga01100 | Metabolic pathways - Gallus gallus (chicken) |
|  |  | gga00051 | Fructose and mannose metabolism - Gallus gallus (chicken) |
|  |  | gga00520 | Amino sugar and nucleotide sugar metabolism - Gallus gallus (chicken) |
| subnet D | *CXCL14* | gga04060 | Cytokine-cytokine receptor interaction - Gallus gallus (chicken) |
|  | *SAR1B* | gga04141 | Protein processing in endoplasmic reticulum - Gallus gallus (chicken) |
| subnet E | *NME1, PGS1, TK1, XYLT2* | gga01100 | Metabolic pathways - Gallus gallus (chicken) |
|  | *GABRA1, GABRG2, GIPR* | gga04080 | Neuroactive ligand-receptor interaction - Gallus gallus (chicken) |
|  | *CANT1, NME1, TK1* | gga00240 | Pyrimidine metabolism - Gallus gallus (chicken) |
|  | *GRB2, SOCS3* | gga04910 | Insulin signaling pathway - Gallus gallus (chicken) |
|  | *CANT1, NME1* | gga00230 | Purine metabolism - Gallus gallus (chicken) |
|  | *NUP85, SUMO2* | gga03013 | RNA transport - Gallus gallus (chicken) |
|  | *GRB2, SUMO2* | gga04630 | Jak-STAT signaling pathway - Gallus gallus (chicken) |
|  | *GRB2* | gga04540 | Gap junction - Gallus gallus (chicken) |
|  |  | gga04320 | Dorso-ventral axis formation - Gallus gallus (chicken) |
|  |  | gga05161 | Hepatitis B - Gallus gallus (chicken) |
|  |  | gga04012 | ErbB signaling pathway - Gallus gallus (chicken) |
|  |  | gga04012 | ErbB signaling pathway - Gallus gallus (chicken) |
|  |  | gga04510 | Focal adhesion - Gallus gallus (chicken) |
|  |  | gga04650 | Natural killer cell mediated cytotoxicity - Gallus gallus (chicken) |
|  |  | gga04912 | GnRH signaling pathway - Gallus gallus (chicken) |
|  |  | gga04010 | MAPK signaling pathway - Gallus gallus (chicken) |
|  | *GGA3* | gga04142 | Lysosome - Gallus gallus (chicken) |
|  | *XYLT2* | gga00534 | Glycosaminoglycan biosynthesis - heparan sulfate / heparin - Gallus gallus (chicken) |
|  |  | gga00532 | Glycosaminoglycan biosynthesis - chondroitin sulfate / dermatan sulfate - Gallus gallus |
|  | *SOCS3* | gga05168 | Herpes simplex infection - Gallus gallus (chicken) |
|  |  | gga04120 | Ubiquitin mediated proteolysis - Gallus gallus (chicken) |
|  |  | ga04920 | Adipocytokine signaling pathway - Gallus gallus (chicken) |
|  |  | gga05164 | Influenza A - Gallus gallus (chicken) |
|  | *TOB1* | gga03018 | RNA degradation - Gallus gallus (chicken) |
|  | *TK1* | gga00983 | Drug metabolism - other enzymes - Gallus gallus (chicken) |
|  | *PGS1* | gga00564 | Glycerophospholipid metabolism - Gallus gallus (chicken) |
|  | *MRPS7* | gga03010 | Ribosome - Gallus gallus (chicken) |
| subnet F | *PIK3CA* | gga04620 | Toll-like receptor signaling pathway - Gallus gallus (chicken) |
|  |  | gga00562 | Inositol phosphate metabolism - Gallus gallus (chicken) |
|  |  | gga04210 | Apoptosis - Gallus gallus (chicken) |
|  |  | gga04810 | Regulation of actin cytoskeleton - Gallus gallus (chicken) |
|  |  | gga04650 | Natural killer cell mediated cytotoxicity - Gallus gallus (chicken) |
|  |  | gga04910 | Insulin signaling pathway - Gallus gallus (chicken) |
|  |  | gga05161 | Hepatitis B - Gallus gallus (chicken) |
|  |  | gga04012 | ErbB signaling pathway - Gallus gallus (chicken) |
|  |  | gga04150 | mTOR signaling pathway - Gallus gallus (chicken) |
|  |  | gga04630 | Jak-STAT signaling pathway - Gallus gallus (chicken) |
|  |  | gga04370 | VEGF signaling pathway - Gallus gallus (chicken) |
|  |  | gga04914 | Progesterone-mediated oocyte maturation - Gallus gallus (chicken) |
|  |  | gga04510 | Focal adhesion - Gallus gallus (chicken) |
|  |  | gga04070 | Phosphatidylinositol signaling system - Gallus gallus (chicken) |
|  |  | gga05164 | Influenza A - Gallus gallus (chicken) |
| subnet G | *BMPR1A* | gga04060 | Cytokine-cytokine receptor interaction - Gallus gallus (chicken) |
|  |  | gga04350 | TGF-beta signaling pathway - Gallus gallus (chicken) |
