# Supplementary material for: Epistatic Effects on Abdominal Fat Content in Chickens: Results from a Genome-Wide SNP-SNP Interaction Analysis
Source: PLoS One. 2013 Dec 5;8(12):e81520. doi: 10.1371/journal.pone.0081520 (PMC3855290; doi:10.1371/journal.pone.0081520)
Supplement: Table S5 — Results of the genome-wide association study of significantly interacting SNPs. The threshold of GWAS is P<2.7×10^−7^ by a Bonferroni 5% correction. (DOC) [file pone.0081520.s005.doc]

**Table S5. Results of the genome-wide association study of significantly interacting SNPs.** The threshold of GWAS is P < 2.7×10^-7^ by a Bonferroni 5% correction.

| GGA | Locus | Test | P_value | Contribution rate (%) |
| --- | --- | --- | --- | --- |
| 0 | GGaluGA194739 | a | 3.56×10^-4^ | 0.4853 |
| 0 | GGaluGA194739 | d | 1.18×10^-1^ | 0.1800 |
| 1 | Gga_rs13749637 | a | 7.12×10^-7^ | 1.0114 |
| 1 | Gga_rs13749637 | d | 2.22×10^-1^ | 0.2061 |
| 1 | Gga_rs15227054 | a | 1.08×10^-6^ | 0.9784 |
| 1 | Gga_rs15227054 | d | 1.95×10^-1^ | 0.2176 |
| 1 | GGaluGA012915 | a | 4.43×10^-3^ | 0.2913 |
| 1 | GGaluGA012915 | d | 1.44×10^-1^ | 0.1440 |
| 1 | Gga_rs13866305 | d | 8.75×10^-1^ | 0.0017 |
| 1 | Gga_rs13866305 | a | 9.67×10^-1^ | 0.0001 |
| 1 | GGaluGA060937 | a | 2.22×10^-3^ | 0.3668 |
| 1 | GGaluGA060937 | d | 5.13×10^-1^ | 0.0505 |
| 2 | GGaluGA146662 | a | 3.15E-03 | 0.2991 |
| 2 | GGaluGA146662 | d | 9.76×10^-1^ | 0.0008 |
| 2 | Gga_rs16026770 | a | 2.61×10^-3^ | 0.3869 |
| 2 | Gga_rs16026770 | d | 4.38×10^-1^ | 0.0916 |
| 2 | Gga_rs14214495 | a | 1.54×10^-1^ | 0.0800 |
| 2 | Gga_rs14214495 | d | 2.30×10^-1^ | 0.1004 |
| 3 | Gga_rs16228738 | a | 4.00×10^-3^ | 0.3568 |
| 3 | Gga_rs16228738 | d | 6.68×10^-1^ | 0.0617 |
| 3 | Gga_rs14319575 | a | 1.96×10^-2^ | 0.2093 |
| 3 | Gga_rs14319575 | d | 3.54×10^-1^ | 0.0655 |
| 3 | Gga_rs16222762 | d | 8.44×10^-2^ | 0.2076 |
| 3 | Gga_rs16222762 | a | 1.56×10^-1^ | 0.0818 |
| 3 | Gga_rs13717259 | a | 6.94×10^-4^ | 0.4036 |
| 3 | Gga_rs13717259 | d | 1.21×10^-1^ | 0.1600 |
| 3 | Gga_rs14340790 | a | 2.48×10^-6^ | 0.8047 |
| 3 | Gga_rs14340790 | d | 3.62×10^-1^ | 0.0714 |
| 3 | Gga_rs16254447 | a | 1.64×10^-5^ | 0.6895 |
| 3 | Gga_rs16254447 | d | 7.76×10^-1^ | 0.0272 |
| 3 | Gga_rs14341204 | a | 2.26×10^-5^ | 0.6559 |
| 3 | Gga_rs14341204 | d | 4.85×10^-1^ | 0.0471 |
| 3 | Gga_rs14341224 | a | 1.64×10^-5^ | 0.6895 |
| 3 | Gga_rs14341224 | d | 7.76×10^-1^ | 0.0272 |
| 3 | Gga_rs14341242 | a | 1.29×10^-5^ | 0.7092 |
| 3 | Gga_rs14341242 | d | 8.58×10^-1^ | 0.0271 |
| 3 | Gga_rs14341255 | a | 1.29×10^-5^ | 0.7092 |
| 3 | Gga_rs14341255 | d | 8.58×10^-1^ | 0.0271 |
| 3 | GGaluGA216762 | a | 1.79×10^-5^ | 0.6765 |
| 3 | GGaluGA216762 | d | 5.57×10^-1^ | 0.0406 |
| 3 | Gga_rs14368109 | d | 5.23×10^-1^ | 0.0273 |
| 3 | Gga_rs14368109 | a | 6.50×10^-1^ | 0.0068 |
| 3 | Gga_rs14368127 | a | 5.94×10^-1^ | 0.0094 |
| 3 | Gga_rs14368127 | d | 6.52×10^-1^ | 0.0136 |
| 3 | Gga_rs14380677 | a | 4.30×10^-1^ | 0.0219 |
| 3 | Gga_rs14380677 | d | 6.85×10^-1^ | 0.0111 |
| 3 | Gga_rs16306728 | d | 2.05×10^-1^ | 0.1072 |
| 3 | Gga_rs16306728 | a | 4.64×10^-1^ | 0.0180 |
| 3 | GGaluGA231041 | a | 4.76×10^-1^ | 0.0182 |
| 3 | GGaluGA231041 | d | 5.96×10^-1^ | 0.0190 |
| 3 | Gga_rs14388313 | a | 9.47×10^-5^ | 0.6450 |
| 3 | Gga_rs14388313 | d | 2.18×10^-1^ | 0.1812 |
| 3 | GGaluGA236122 | a | 4.53×10^-4^ | 0.4073 |
| 3 | GGaluGA236122 | d | 3.03×10^-1^ | 0.0690 |
| 3 | Gga_rs14402423 | a | 4.53×10^-4^ | 0.4073 |
| 3 | Gga_rs14402423 | d | 3.03×10^-1^ | 0.0690 |
| 4 | Gga_rs15480969 | a | 2.49×10^-5^ | 0.6889 |
| 4 | Gga_rs15480969 | d | 5.66×10^-1^ | 0.061 |
| 5 | Gga_rs14521876 | a | 1.53×10^-1^ | 0.0774 |
| 5 | Gga_rs14521876 | d | 2.68×10^-1^ | 0.0844 |
| 6 | Gga_rs13561344 | a | 5.38×10^-5^ | 0.6311 |
| 6 | Gga_rs13561344 | d | 1.27×10^-2^ | 0.4377 |
| 6 | Gga_rs14560750 | a | 5.17×10^-5^ | 0.5800 |
| 6 | Gga_rs14560750 | d | 1.49×10^-1^ | 0.1413 |
| 7 | GGaluGA317680 | a | 1.16×10^-3^ | 0.4473 |
| 7 | GGaluGA317680 | d | 6.42×10^-1^ | 0.0680 |
| 8 | GGaluGA333545 | a | 4.66×10^-1^ | 0.0197 |
| 8 | GGaluGA333545 | d | 8.82×10^-1^ | 0.0019 |
| 8 | Gga_rs14658668 | a | 4.75×10^-1^ | 0.0190 |
| 8 | Gga_rs14658668 | d | 8.79×10^-1^ | 0.0020 |
| 8 | Gga_rs16650878 | a | 4.68×10^-1^ | 0.0198 |
| 8 | Gga_rs16650878 | d | 8.82×10^-1^ | 0.0019 |
| 9 | Gga_rs16674724 | a | 5.53×10^-7^ | 0.9779 |
| 9 | Gga_rs16674724 | d | 1.46×10^-1^ | 0.2004 |
| 10 | GGaluGA066690 | a | 9.22×10^-4^ | 0.4591 |
| 10 | GGaluGA066690 | d | 1.93×10^-2^ | 0.4117 |
| 10 | GGaluGA066877 | a | 4.45×10^-4^ | 0.4561 |
| 10 | GGaluGA066877 | d | 1.30×10^-1^ | 0.1622 |
| 10 | GGaluGA069801 | d | 7.76×10^-2^ | 0.2081 |
| 10 | GGaluGA069801 | a | 3.27×10^-1^ | 0.0353 |
| 10 | Gga_rs15583507 | a | 3.98×10^-3^ | 0.3975 |
| 10 | Gga_rs15583507 | d | 2.88×10^-1^ | 0.1869 |
| 10 | Gga_rs14009265 | a | 3.49×10^-3^ | 0.3630 |
| 10 | Gga_rs14009265 | d | 7.92×10^-1^ | 0.0509 |
| 10 | GGaluGA071224 | a | 1.31×10^-3^ | 0.3408 |
| 10 | GGaluGA071224 | d | 9.33×10^-1^ | 0.0005 |
| 10 | Gga_rs15589655 | a | 1.58×10^-4^ | 0.5567 |
| 10 | Gga_rs15589655 | d | 2.13×10^-1^ | 0.1336 |
| 13 | Gga_rs15683090 | a | 1.56×10^-5^ | 0.6509 |
| 13 | Gga_rs15683090 | d | 1.83×10^-1^ | 0.1196 |
| 13 | Gga_rs14988623 | d | 1.46×10^-2^ | 0.3928 |
| 13 | Gga_rs14988623 | a | 7.32×10^-2^ | 0.1038 |
| 13 | Gga_rs16002106 | a | 9.86×10^-4^ | 0.4390 |
| 13 | Gga_rs16002106 | d | 4.80×10^-1^ | 0.0703 |
| 13 | GGaluGA097211 | a | 1.78×10^-4^ | 0.6245 |
| 13 | GGaluGA097211 | d | 6.21×10^-1^ | 0.1319 |
| 13 | GGaluGA097233 | a | 3.07×10^-3^ | 0.3532 |
| 13 | GGaluGA097233 | d | 6.53×10^-1^ | 0.0411 |
| 14 | Gga_rs15717370 | a | 4.72×10^-2^ | 0.1398 |
| 14 | Gga_rs15717370 | d | 8.42×10^-1^ | 0.0037 |
| 14 | Gga_rs14068999 | a | 4.72×10^-2^ | 0.1398 |
| 14 | Gga_rs14068999 | d | 8.42×10^-1^ | 0.0037 |
| 14 | Gga_rs15718248 | a | 4.53×10^-2^ | 0.1487 |
| 14 | Gga_rs15718248 | d | 7.77×10^-1^ | 0.0088 |
| 14 | GGaluGA101229 | a | 6.61×10^-3^ | 0.2812 |
| 14 | GGaluGA101229 | d | 8.56×10^-2^ | 0.2054 |
| 14 | Gga_rs14075705 | a | 8.81×10^-4^ | 0.4124 |
| 14 | Gga_rs14075705 | d | 2.55×10^-1^ | 0.0972 |
| 18 | Gga_rs14416916 | d | 1.15×10^-2^ | 0.4221 |
| 18 | Gga_rs14416916 | a | 3.88×10^-1^ | 0.0263 |
| 18 | Gga_rs15469971 | d | 1.91×10^-3^ | 0.6374 |
| 18 | Gga_rs15469971 | a | 2.71×10^-1^ | 0.0430 |
| 18 | Gga_rs10729280 | d | 6.44×10^-2^ | 0.2276 |
| 18 | Gga_rs10729280 | a | 2.98×10^-1^ | 0.0392 |
| 18 | Gga_rs13569377 | d | 1.61×10^-1^ | 0.1315 |
| 18 | Gga_rs13569377 | a | 6.52×10^-1^ | 0.0071 |
| 20 | Gga_rs14272866 | a | 1.77×10^-1^ | 0.0735 |
| 20 | Gga_rs14272866 | d | 2.67×10^-1^ | 0.0875 |
| 20 | Gga_rs14276105 | a | 4.39×10^-9^ | 1.4599 |
| 20 | Gga_rs14276105 | d | 5.65×10^-1^ | 0.2391 |
| 23 | Gga_rs13622160 | a | 1.06×10^-2^ | 0.2377 |
| 23 | Gga_rs13622160 | d | 7.97×10^-1^ | 0.0085 |
| 23 | GGaluGA188871 | a | 2.88×10^-2^ | 0.1729 |
| 23 | GGaluGA188871 | d | 3.21×10^-1^ | 0.0680 |
| 23 | Gga_rs14290610 | a | 6.38×10^-4^ | 0.4494 |
| 23 | Gga_rs14290610 | d | 1.33×10^-1^ | 0.1707 |
| 27 | Gga_rs14303341 | a | 2.72×10^-3^ | 0.4155 |
| 27 | Gga_rs14303341 | d | 5.23×10^-1^ | 0.1207 |
| Z. | Gga_rs14748835 | a | 1.04×10^-3^ | 0.4448 |
| Z. | Gga_rs14748835 | d | 9.16×10^-1^ | 0.0458 |
| Z. | Gga_rs16094710 | a | 1.04×10^-3^ | 0.4448 |
| Z. | Gga_rs16094710 | d | 9.16×10^-1^ | 0.0458 |
| Z. | Gga_rs16758057 | a | 1.40×10^-3^ | 0.4221 |
| Z. | Gga_rs16758057 | d | 9.79×10^-1^ | 0.0439 |
| Z. | Gga_rs15991936 | a | 9.74×10^-3^ | 0.3083 |
| Z. | Gga_rs15991936 | d | 5.25×10^-1^ | 0.0946 |
